# Enhancing IPTp-SP uptake: Community and stakeholder recommendations for improving access and utilisation – insights from a study in Bayelsa-Nigeria

**DOI:** 10.5281/zenodo.15351243

**Published:** 2025-05-06

**Authors:** Patricia Ogba, Andrea Baumann, Tunde Alabi, Norm Archer, Joshua Eniojukan, Bonny Ibhawoh, Deborah D. DiLiberto

**Affiliations:** 1 Global Health Office, Faculty of Health Sciences, McMaster University, Main St. W, Hamilton, Ontario, Canada.; 2 Department of Sociology, University of Lagos, Akoka, Nigeria.; 3 Degroote School of Business, McMaster University, Main St. W, Hamilton, Ontario, Canada.; 4 Department of Clinical Pharmacy, Niger-Delta University, Wilberforce Island, Bayelsa, Nigeria.; 5 Department of History, McMaster University, Main St. W, Hamilton, Ontario, Canada.

## Abstract

**Background:**

Malaria remains a major global health challenge, disproportionately affecting pregnant women and children. In Nigeria, malaria in pregnancy contributes to 70.5% of maternal morbidity and 41.1% of maternal mortality. Recognising these risks, the World Health Organization recommends intermittent preventive treatment with sulfadoxine-pyrimethamine (IPTp-SP) as a key strategy for malaria in pregnancy prevention. However, despite its proven effectiveness, pregnant women’s uptake of IPTp-SP remains unacceptably low. This study presents participant-driven recommendations to enhance IPTp-SP uptake, structured within the socio-ecological framework.

**Materials and Methods:**

This study employed an exploratory descriptive qualitative approach to examine the community-level contextual factors influencing IPTp-SP uptake. Data were collected from 53 participants in two communities in Bayelsa, Nigeria. Individual interviews were conducted with 17 key stakeholders (spouses, mothers-in-law, religious leaders, community leaders, and traditional birth attendants) and 6 focus group discussions with 36 pregnant women. Data management and coding were conducted using NVivo 14 QSR International software, following an inductive-deductive thematic analysis approach.

**Results:**

Participants proposed multi-level interventions to address barriers to IPTp-SP uptake at the individual, interpersonal, community, and healthcare system levels. Key recommendations include: Community-wide education campaigns to raise awareness of IPTp-SP’s benefits; comprehensive training for healthcare providers to enhance their knowledge and prescription of IPTp-SP; integration of traditional birth attendants into the formal healthcare system; community-level distribution of IPTp-SP to improve access for pregnant women who do not attend antenatal care; government intervention to ensure the functionality of health centers; addressing workforce shortages, and guaranteeing a consistent supply of IPTp-SP.

**Conclusion:**

These evidence-based, participant-driven recommendations offer a holistic and scalable strategy to improve pregnant women’s uptake of IPTp-SP in Nigeria and other malaria-endemic regions. Implementing these recommendations can strengthen malaria prevention efforts, improve maternal and child health outcomes, and support broader public health initiatives.

## Introduction

Malaria poses a significant global health challenge, notably affecting children and expectant mothers. Despite a consistent decline in malaria-related deaths, from 897,000 in 2000 to 568,000 in 2019, the number increased to 619,000 in 2021, attributed to disruptions caused by the COVID-19 pandemic [1]. According to the World Malaria Report (2024), an estimated 597,000 malaria deaths occurred in 2023. That same year, approximately 263 million malaria cases were reported globally—an increase of 11 million cases compared to 2022 [2]. The World Health Organisation’s African Region continues to bear the highest burden, accounting for an estimated 94% of global malaria cases and approximately 95% of malaria-related deaths in 2023 [2]. Pregnant women, particularly during their first and second pregnancies, face heightened vulnerability due to reduced immunity associated with pregnancy [3-6]. Malaria in pregnancy (MiP) significantly contributes to maternal mortality and unfavourable pregnancy outcomes in Nigeria [5,7]. Malaria is responsible for nearly half of all reported diseases and contributes to 15% of anaemia among pregnant women [8,9]. MiP causes 70.5% of maternal morbidity and 41.1% of maternal mortality [8-11]. Between 2017 and 2019, the number of pregnant women affected by malaria in Nigeria reduced from around 480,000 to approximately 373,000 [12]. Despite the decline in malaria morbidity among expectant mothers, the remaining number is alarming and warrants urgent attention. Among infants, MiP results in 5-14% of cases of low birth weight (LBW), with approximately 30% being preventable [8-11]. MiP challenges attaining Sustainable Development Goal 3, which targets maternal mortality reduction and malaria eradication by 2030 [9,13,14]. Recognising the adverse effects of malaria on pregnant women and their unborn children, the WHO recommends a comprehensive approach, among which is the use of intermittent preventive treatment with sulfadoxine-pyrimethamine (IPTp-SP) [9,15-17].

IPTp-SP is a critical malaria prevention strategy involving the monthly administration of an entire antimalarial course from the second trimester until delivery. Each dose consists of three tablets containing 500 mg of sulfadoxine and 25 mg of pyrimethamine. The medication can be taken with or without food but should not be used concurrently with daily folic acid supplementation or co-trimoxazole prophylaxis ![6,15,17,18].

In 2004, to reduce the MiP burden, particularly in malaria-endemic areas, the WHO first recommended that all pregnant women receive two doses of IPTp-SP [4,19]. In 2012, the WHO updated and revised the policy, recommending pregnant women take more than two doses of IPTp-SP. The initial dose should be given as early as feasible in the second trimester, with subsequent doses provided at one-month intervals until delivery [15,19,20]. Nigeria adopted IPTp-SP as a strategy in 2005 and updated it in 2014 to align with the WHO recommendations [8,16,20,01]. Under the Nigerian malaria treatment protocol, IPTp-SP is provided free of charge during routine antenatal care (ANC) visits as part of the directly observed treatment (DOT) strategy [6,16,22].

Despite global advances in antenatal care uptake, ANC utilisation in Nigeria remains notably low [23-25]. Significant rural–urban disparities persist, with women in urban areas approximately 3.5 times more likely to access ANC services compared to their rural counterparts [24]. Furthermore, most women who attend ANC do not meet the WHO’s recommended minimum of eight contacts, with only about one-fifth of mothers achieving this benchmark [23-25]. Among the states with particularly poor ANC completion rates is Bayelsa, identified as having high levels of incomplete visits [25].

In addition to limited ANC attendance, the uptake of IPTp-SP remains suboptimal despite its proven benefits [3,7-9,13,17-21], highlighting the need for targeted interventions to improve accessibility and adherence. Even among women who attend at least four ANC visits, over two-thirds still receive fewer than the recommended three doses of IPTp-SP [20,28]. This shortfall is further exacerbated by frequent stock-outs of IPTp-SP in health facilities, which significantly constrain its availability and access [19,28,29]. These consistent stock-outs represent a critical barrier to adhering to the WHO malaria prevention guidelines during pregnancy [8].

To our knowledge, no study has specifically explored strategies for enhancing pregnant women's IPTp-SP uptake through community and stakeholder recommendations using the Appreciative Inquiry lens and socioecological framework. As part of a broader study examining community-level contextual factors influencing IPTp-SP utilisation among pregnant women in Nigeria, this paper presents participant-driven recommendations for improving uptake structured within the socio-ecological framework.

## Conceptual Framework

This study utilised the Appreciative Inquiry approach and the Socio-Ecological Model. The Appreciative Inquiry (AI) approach is a strengths-based framework that explores what works well rather than focusing solely on problems [31]. AI informed both the design of the interview guide and the interpretation of results by centring community-driven solutions. The guide included positively framed questions that invited participants to consider how pregnant women could be better supported to increase IPTp-SP uptake. This approach enabled participants to identify both barriers and context-specific strategies for improvement.

The Socio-Ecological Model (SEM), initially introduced by Urie Bronfenbrenner in the 1970s as a framework for understanding human development, was formalised as a theory in the 1980s [32]. It highlights the complex interplay between individual, community, and environmental factors—encompassing individual, interpersonal, community, organisational, and public policy factors—that shape health behaviours [2,31,32] at different levels:

1. **Individual level**: Focuses on personal factors like knowledge, attitudes, beliefs, genetics, and history that influence behaviour. For example, a pregnant woman’s awareness of IPTp-SP and its benefits.
2. **Interpersonal level**: Examines relationships with family, friends, and social networks that affect health behaviours. For example, the role of mothers-in-law or partners in encouraging or discouraging IPTp-SP use.
3. **Community Level**: Considers structured communities such as neighbourhoods where social interactions occur. For example, community norms and support systems influencing attitudes toward ANC services and IPTp-SP.
4. **Organisational/Institutional level**: Involves practices and structures within institutions like healthcare facilities that impact health. For example, availability of resources, trained providers, and consistent IPTp-SP supply.
5. **Policy/Societal level**: Includes policies that shape health behaviours and service access. For example, policies on free IPTp-SP distribution, government commitment to drug supply, and removing user fees for maternal health services.

**Figure 1:**
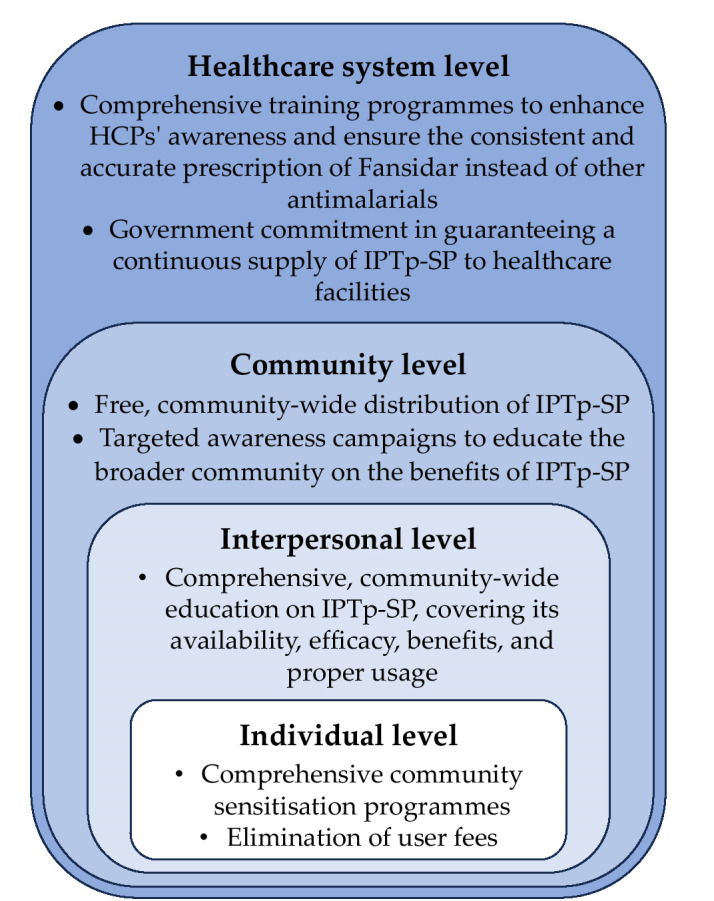
An adapted socioecological framework showing participants’ recommendations on improving pregnant women’s IPTp-SP uptake at the individual, interpersonal, community, and healthcare system levels.

This paper will focus on the first four levels: individual, interpersonal, community, and organisational (Figure 1). The organisational level refers to the healthcare system. Applying the socio-ecological framework to improve IPTp-SP uptake provides a holistic approach that acknowledges these multifaceted influences on pregnant women’s health decisions. By integrating community and stakeholder-driven recommendations, this study offers practical insights for policymakers, healthcare providers, and community leaders to design targeted interventions. Implementing these strategies could enhance accessibility, awareness, and adherence to IPTp-SP, ultimately mitigating malaria-related maternal and neonatal health risks in Nigeria.

## Materials and Methods

This study was part of a broader exploratory descriptive qualitative research investigating community-level contextual factors influencing the uptake of preventive treatments for malaria during pregnancy. It was guided by Appreciative Inquiry, an innovative problem-solving approach focused on strengths rather than deficits [31]. As such, we engaged participants in positive discussions to identify support strategies for enhancing pregnant women’s IPTp-SP uptake.

### Study location and population

This study took place in Yenaka and Sabagreia, two rural communities in Bayelsa State, Nigeria. The region's waterlogged terrain fosters year-round mosquito breeding, sustaining persistent malaria transmission [34,35]. Malaria remains a leading cause of death, exacerbated by limited healthcare access, reliance on traditional medicine, and self-medication due to poverty and rugged terrain [34,37]. Yenaka has one partially functioning health center with inadequate equipment, essential drugs, and emergency facilities [38]. Sabagreia has two poorly equipped cottage hospitals run by community health extension workers. Neither facility operates 24/7.

The study engaged pregnant women and stakeholders, including spouses, mothers-in-law, religious leaders, community leaders, and traditional birth attendants (TBAs), to identify strategies for enhancing IPTp-SP utilisation. Pregnant women were conveniently sampled, and stakeholders were purposely selected based on their community roles. Participants were identified and selected with the assistance of four hired research assistants. Pregnant women were recruited directly from TBA clinics in the two target communities. The partners of the pregnant women interested in participating in the study were also recruited. In addition, key stakeholders—including mothers-in-law, community leaders, religious leaders, and TBAs—were purposefully selected based on their roles and influence within the community. This approach ensured that the sample reflected both the direct experiences of pregnant women and the perspectives of those influential in shaping community health practices.

### Interviews and focus group discussions

A total of 17 stakeholder interviews and 6 focus group discussions (FGDs) with pregnant women were conducted across the two communities. In Sabagreia and Yenaka, 3 FGDs were held in each, with 6 pregnant women per group. In-depth interviews (IDIs) were conducted with 4 partners, 4 mothers-in-law, 2 community leaders, 4 religious leaders, and 3 TBAs.

Participants ranged from 18-38 years old, with age diversity having no impact on discussions. Of the 36 pregnant women, 50% had no formal education, 35% completed primary school, and 15% reached secondary education. Less than half (42%) were married. Though residing in the same communities, they represented various ethnic groups, including Eppie, Atisa, Ijaw, Isoko, Ikorobasi, Akwa-Ibom, Kolokuma, and Ikot-Ekpene. All identified as Christians, with most (96%) working as farmers and 4% as housewives.

We adapted Nyaaba *et al.*'s interview guide [6] for data collection, piloting it with 10 pregnant women from two contiguous communities. Their responses were excluded from the main study. Due to time constraints, stakeholder interviews were not piloted. The pilot confirmed the questions were well-formulated and culturally sensitive, requiring only language adaptation. Women preferred Ijaw (local dialect) or pidgin English (colloquial English), so interviews were conducted accordingly. Spouses, TBAs, and mothers-in-law opted for Ijaw, while religious and community leaders chose English. The guide, originally in English, was not translated due to a lack of suitable translators. Instead, research assistants interpreted questions as needed. The guide included open-ended questions.

We collected data through face-to-face FGDs and IDIs between 9^th^ of May and 29^th^ of June, 2022. This research involved 17 IDIs with stakeholders and 6 FGDs, each comprising 6 pregnant women, totalling 36. Interviews with stakeholders lasted 30-45 minutes, and FGDs with pregnant women lasted 1-1.5 hrs. Interviews continued until data saturation was reached.

### Data analysis

All IDIs and FGDs were interpreted and transcribed directly into English rather than verbatim in the original Ijaw dialect or pidgin English. Due to the limited availability of transcriptionists fluent in Ijaw, the research assistants conducted real-time interpretation during interviews and transcribed them in English. The exact process was followed for interviews conducted in pidgin English to ensure consistency. The interview transcripts were managed and analysed using NVivo 12 qualitative analysis software by QSR International.

We used a hybrid deductive-inductive thematic analysis for the larger study to balance our research objectives with an openness to emerging insights. The analysis began with a deductive coding framework informed by the study’s research questions. Initial themes included knowledge of MiP, IPTp-SP use, prevention strategies, willingness to use IPTp-SP, community influences (e.g., culture, finance), and needed support systems (reported in this paper). Concurrently, we applied an inductive approach to capture unanticipated themes from the data. For example, pregnant women’s attitudes toward IPTp-SP emerged organically, adding depth to our understanding. The codebook was refined iteratively as new insights surfaced. This hybrid approach allowed for a comprehensive and flexible analysis grounded in theory and participant experience [39].

### Position statement

The lead author is a Nigerian researcher who completed her secondary and undergraduate education in Nigeria. As a registered nurse and midwife with >9 years of experience in government and private hospitals, she has firsthand knowledge of malaria's impact on pregnant women and their unborn babies. This professional experience informed her interest in the study. The author engaged in reflexive practices throughout the research to mitigate potential bias stemming from her healthcare background. She conducted member checking by sharing key findings from the thematic analysis, through the study research assistants, to the study participants for review and honing to ensure her interpretations accurately reflected their perspectives. The four research assistants who conducted interviews, took notes, and handled the transcription are from the study communities. Their insider status fostered participant trust but also required reflexive attention. Regular debriefings were held with the research assistants to reflect on how their position might influence data collection and interpretation, helping to ensure findings remained grounded in participants’ lived experiences.

### Trustworthiness and rigour

Trustworthiness in qualitative research, encompassing credibility, dependability, confirmability, and transferability, ensures research quality [39-40]. This study provided credibility through member checking and triangulation. We addressed transferability by situating findings within the existing literature. Further, a community member cross-checked audio recordings with interview transcripts, and the codes developed for analysis by PO were validated by a co-author (T.A.), adding another layer of quality assurance.

### Ethical considerations

Ethical approval was obtained from the Hamilton Integrated Research Ethics Board [HiREB#: 14382], Canada, and the Bayelsa State Ministry of Health, Nigeria, along with letters of permission from the leaders of the studied communities. Stringent ethical measures, including informed consent and confidentiality, were followed, allowing participants to opt out at any stage. Research assistants explained the study's purpose and procedure, originally written in English, to pregnant women without formal education in their local dialects. They verbally agreed to participate and endorsed the consent forms with thumbprints. A similar process was followed for spouses, mothers-in-law, and TBAs without formal education. However, a distinct procedure was employed for the community and religious leaders. They independently read the document containing the study purpose and procedure, sought clarifications by posing questions, and subsequently gave written consent and affixed their signatures. Informed consent was obtained verbally and in written format before each interview. Interviews were recorded with consent and stored securely. COVID-19 precautions, like social distancing and mask use, were strictly adhered to, in line with Nigeria's Ministry of Health guidelines.

## Results

This manuscript draws from a broader study with predefined themes, including understanding of MiP, IPTp-SP use, MiP prevention and treatment strategies, willingness to adopt IPTp-SP, community influences (tradition, ethnicity, culture, history, finances) on IPTp-SP uptake, and support needed to improve pregnant women’s IPTp-SP uptake. An unexpected theme, pregnant women's attitudes, also emerged during analysis.

Focusing on the theme ‘support needed to improve pregnant women’s IPTp-SP uptake’, this article applies the socio-ecological model to present participants’ recommendations on improving pregnant women’s IPTp-SP uptake at the individual, interpersonal, community, and healthcare system levels.

### Individual/intrapersonal level

This study identified several intrapersonal barriers affecting the uptake of IPTp-SP among pregnant women. Key factors include inadequate knowledge of IPTp-SP, negative perceptions about its efficacy, poor ANC attendance, reliance on TBAs and herbal remedies, and financial constraints. Many participants demonstrated limited awareness and understanding of IPTp-SP's effectiveness and benefits in preventing MiP. Several participants revealed that their first exposure to information about IPTp-SP's protective role came from participating in this study. Additionally, some pregnant women who participated in this study, concerned about potential adverse effects on their pregnancy outcomes, often refrain from using the drug. ANC attendance was low, with only a few pregnant women reporting visits to healthcare facilities for proper maternal care. Instead, many preferred seeking treatments from chemists and pharmacies, while others relied on herbal mixtures provided by TBAs. When asked about their treatment choice, those who visited chemists cited affordability and proximity as key reasons. Meanwhile, women who opted for herbal remedies often did so due to a fear of pharmaceutical drugs and injections. Although pregnant women expressed a strong willingness to take Fan-sidar®, financial barriers posed a challenge. Many recounted experiences of visiting clinics expecting to receive Fansidar® as part of their antenatal care, only to be informed by HCPs that they needed to pay for it or purchase it externally, leading to frustration and limited access.

#### Recommended interventions

First, the need for comprehensive community sensitisation programmes to educate pregnant women and community members about the benefits and availability of IPTp-SP was emphasised. Raising awareness could improve knowledge and encourage greater uptake of preventive treatment:

“*Awareness campaigns or programmes concerning Fansidar®, gather people in the community and sensitise people about the drugs”* (Spouse)

*“…Go into the community, see pregnant women, educate them…”* (Religious leader)

Participants also highlighted the urgent need to refurbish existing healthcare facilities, many of which are in a poor state. Additionally, they recommended constructing well-equipped, adequately staffed hospitals to enhance ANC services and encourage higher attendance among pregnant women:

*“The government should try to keep the health centers in the local communities working and also make good provision of IPTp-SP (Fansidar®) so it will always be available for pregnant women in the community”* (Mother-in-law)

*“…if the government can give us doctors and nursing staff, pregnant women will go for treatment”* (Community leader)

Study participants expressed the importance of educating pharmacy and chemist operators, as these are their first contact points when they need drugs:

*“…people operating pharmacies and chemists should be enlightened about this drug so that they can accurately prescribe it to us whenever we patronise them”* (FGD BC1)

Recognising the continued reliance on TBAs and herbal remedies, participants strongly advocated for the integration of TBAs into the formal healthcare system. This integration would allow TBAs to operate under the supervision of qualified HCPs, ensuring that maternal care practices align with established medical guidelines. They also recommended that the government supply TBAs with Fansidar® and provide them with proper training on its administration. By equipping TBAs with both the knowledge and resources to administer Fansidar® safely, pregnant women who visit them —particularly for traditional massages—would have improved access to malaria prevention. This strategy could bridge the gap between conventional and formal healthcare services, enhancing the overall effectiveness of antenatal care in the community:

*“On behalf of my colleagues and I, I appeal that we should be attached to the health center”* (TBA)

*“They can also provide these tablets to traditional midwives and educate them on how to administer it so that they can give us when we go to them for a massage”* (FGD BC1)

Finally, participants urged the government to guarantee a consistent supply of Fansidar® in clinics, making it both readily available and free of charge for pregnant women attending ANC services. Ensuring free access to Fansidar® could reduce dependence on chemists, where IPTp-SP is often unavailable, thereby improving malaria prevention during pregnancy:

*“The tablet should be readily accessible and available for free of charge in health centres…The drug should not be sold to us… Government should supply it to hospitals so that HCPs will give the drugs to us for free whenever we visit the hospital for ANC”* (FGD BC1)

*“…the drugs should be available at all times”* (Community leader)

### Interpersonal level

The study identified key interpersonal factors, particularly the role of mothers-in-law and broader family dynamics, in shaping pregnant women's healthcare decisions. A striking finding revealed that many pregnant women are inclined to follow advice from their mothers-in-law, parents, and siblings—even if it means forgoing IPTp-SP use when discouraged. For instance, participants emphasised the pivotal influence of mothers-in-law in marriage, recounting how some actively dissuade their daughters-in-law from taking prescribed medications. Instead, these women are brought to herbal homes, where they are given traditional herbal mixtures purported to ease childbirth. While some participants firmly stated that they would neither accept these herbal remedies nor abandon prescribed medications such as Fansidar® due to family influence, a considerable number admitted they would prioritise the local remedy over Fansidar® if their mothers-in-law recommended it. This underscores the profound impact of interpersonal relationships on women's healthcare-seeking behaviours.

#### Recommended interventions

To address this challenge, participants advocated for comprehensive, community-wide education on IPTp-SP, covering its availability, efficacy, benefits, and proper usage. They stressed that such awareness efforts should not be limited to pregnant women alone but should extend to the entire community:

*“…come and do awareness campaign or programme concerning drugs, gather people in the community, and sensitise people about the drug”* (Spouse)

*“…how I wish more enlightenment would be carried out”* (Community leader)

### Community level

The study identified several community-level factors influencing IPTp-SP uptake, including the lack of healthcare facilities, cultural practices, and the influence of religious and community leaders. Many pregnant women attributed the low utilisation of IPTp-SP to limited access to healthcare centers, which subsequently affects the drug's availability. While cultural traditions—such as the use of herbal remedies and specific dietary customs during pregnancy—do not explicitly discourage IPTp-SP use, some women expressed a firm reluctance to take the medication if it conflicted with their cultural beliefs. Notably, half of the pregnant women reported that they would forgo IPTp-SP for malaria prevention if their pastors advised them to do so after prayers. While some participants were willing to challenge their community leaders’ views on IPTp-SP, others stated they would abstain from using the drug if their leaders advised against it.

#### Recommended interventions

To address these challenges, study participants emphasised the urgent need to refurbish existing community health centers and ensure a steady supply of IPTp-SP. Additionally, they advocated for free, community-wide distribution of the drug. Community leaders suggested leveraging the influence of local chiefs to disseminate information on drug distribution schedules, ensuring that pregnant women no longer face barriers due to healthcare facility shortages. Study participants also recommended targeted awareness campaigns to educate the broader community on the benefits of IPTp-SP. They stressed that when pregnant women and key stakeholders understand the importance of the medication, they may be more inclined to prioritise its use over traditional customs. Furthermore, reducing misinformation among influential figures—such as religious leaders—could minimise discouragement against IPTp-SP uptake:

*“…so the government has a role to play in putting our clinics in a good shape…and to provide the drug that will protect our pregnant women from malaria”* (Community Leader)

*“…distributing the drugs within the community without me going to the hospital will improve my uptake”* (FGD BC1)

*“…come and do awareness campaign or programme concerning drugs, gather people in the community, and sensitise people about the drug”* (Spouse)

### Healthcare system level

This study identified several health system challenges that hinder the uptake of IPTp-SP, including inadequate HCPs’ prescription practices, frequent stock-outs of Fansidar®, and user fees. Participants highlighted how HCPs often prescribed alternative antimalarials instead of IPTp-SP during antenatal visits. Additionally, many pregnant women attributed their non-use of IPTp-SP to its frequent unavailability at clinics. Even when the drug was available, they were often required to pay for it or directed to purchase it from external pharmacies, creating further barriers to access.

#### Recommended Interventions

To address these challenges, study participants emphasised the need for comprehensive training programmes to enhance HCPs' awareness and ensure the consistent and accurate prescription of Fansidar® instead of other antimalarials. Participants also underscored the importance of government commitment in guaranteeing a continuous supply of IPTp-SP to healthcare facilities. By ensuring free and uninterrupted availability, the need for pregnant women to purchase the drug externally would be eliminated, ultimately improving IPTp-SP uptake:

*“HCPs should be enlightened about this drug so that they can accurately prescribe it to us whenever we patronise them…”* (FGD BC1)

*“…government should supply it to hospitals so that HCPs will give the drugs to us for free whenever we visit the hospital for ANC”* (FGD BC2)

*“…the drugs should be available at all times”* (Community leader)

## Discussion

This study identified key challenges hindering pregnant women's utilisation of IPTp-SP and has outlined corresponding recommendations across the individual, interpersonal, community, and healthcare system levels. A comprehensive, multilevel approach can significantly enhance maternal and child health outcomes while strengthening malaria prevention efforts.

Study participants strongly advocated for community-wide education campaigns and awareness programmes to combat the widespread lack of awareness and understanding among community members and pregnant women regarding the efficacy and proper use of IPTp-SP. These initiatives would provide crucial information on the availability, benefits, and safety of IPTp-SP, ultimately fostering greater acceptance and uptake. Research [7,41-46] has demonstrated that proper education about IPTp-SP’s benefits on pregnancy outcomes can significantly enhance its utilisation among pregnant women. By addressing these barriers, targeted awareness efforts can be pivotal in promoting informed decision-making and improving maternal health outcomes.

Findings also underscored the critical need for comprehensive education and training for HCPs on IPTp-SP usage, efficacy, and benefits in preventing MiP. Study participants emphasised that well-informed HCPs are more likely to consistently prescribe IPTp-SP and serve as key advocates in educating pregnant women about its importance. This finding has been reported in several studies [3,7,44,47-50]. Participants also highlighted the need to educate pharmacists and chemists in the studied communities, recognising that these individuals often serve as the first point of contact for pregnant women seeking healthcare. Participants stressed that equipping them with accurate knowledge about IPTp-SP’s role in MiP prevention can help ensure that pregnant women receive the correct information and appropriate antimalarial interventions when seeking treatment outside formal healthcare settings. Several studies [28,51,52] have also recommended this.

Although participants across the different SEM levels strongly recommended sensitisation, it may not be sufficient to drive behavioural change. The health belief model has demonstrated that people’s perception of the severity and their susceptibility to certain health conditions matters in whether or not they will engage in preventive behaviours. In addition, even when people are sensitised and understand the importance of preventive behaviours, barriers beyond their immediate control could inhibit their IPTp-SP uptake. Furthermore, knowledge of malaria may not necessarily translate to effective preventive behaviour or attitude [53-54]. While the intervention study of Sano *et al.* [49] in Guinea found that the intervention group had improved malaria knowledge and practice, the attitude did not vary significantly between intervention and control groups. In Nigeria, Adejoh *et al.* [50] reported that the southern women were more educated and knowledgeable than the northern women. However, 51.4% of the latter group used treated nets for their children compared to 27.4% of the former. The lesson here is that sensitisation needs to be combined with other complex social, religious and economic factors that can affect actual behavioural change.

This study highlights the critical role of TBAs as the first point of contact for many pregnant women seeking ANC services. Previous studies [55-58] have documented pregnant women’s high patronage of TBAs, particularly in rural areas. Participants noted that TBAs are highly preferred due to their massage services, which are valued as part of traditional maternal care. However, a key concern raised was that TBAs do not typically prescribe IPTp-SP for malaria prevention, limiting access to this essential intervention. To address this gap, study participants recommended integrating TBAs into the formal healthcare system through structured training programmes led by qualified HCPs. This initiative would equip TBAs with the necessary knowledge and resources to administer IPTp-SP effectively. Additionally, participants advocated for directly supplying TBAs with IPTp-SP, enabling them to provide it during routine massage sessions. Incorporating TBAs into the healthcare system—through training, supervision, and access to IPTp-SP—can significantly improve IPTp-SP uptake and enhance maternal and child health outcomes, particularly in underserved communities. This approach aligns with global health recommendations, including those set forth by the WHO and the Alma-Ata Declaration (1978), emphasising collaboration between TBAs and HCPs in delivering maternal healthcare at the primary healthcare level [52]. Prior research [5,55] also supports this integration to strengthen maternal healthcare services in resource-limited settings.

Findings from our study highlight the need for community-driven interventions to improve IPTp-SP access, particularly for pregnant women who do not attend ANC and, consequently, miss out on malaria prevention. Pregnant women, TBAs, mothers-in-law, and religious leaders advocated for community-level distribution of IPTp-SP, ensuring that those outside the formal healthcare system can still receive the medication. These interventions involve training and mobilising community-based SP distributors to provide IPTp-SP outside traditional healthcare settings. By expanding IPTp-SP distribution to non-healthcare settings, such as community gatherings or religious events, these measures aim to reach pregnant women who do not regularly attend ANC clinics [43,51]. While community-based distribution of IPTp-SP offers a promising strategy to increase access, it must not detract from the critical role of ANC attendance. ANC serves as a vital platform for delivering comprehensive maternal health services, including early detection and management of pregnancy-related complications, nutritional supplementation (e.g., folic acid and iron), immunisations, and prevention of mother-to-child transmission of infections such as HIV/AIDS [22,23,25]. It also provides structured opportunities for health education, counselling, and screening for high-risk conditions, all of which contribute to improved maternal and perinatal outcomes [22,23,25,26]. Therefore, any recommendation, such as community distribution of IPTp-SP, should be embedded within a holistic maternal health framework. This approach ensures that such strategies complement, rather than replace, ANC services while promoting regular facility-based care to optimise maternal and newborn health outcomes.

In response to the poor state of healthcare facilities, shortage of trained HCPs, and inconsistent availability of IPTp-SP in the studied communities, participants emphasised the urgent need for sustained government commitment to healthcare delivery and malaria prevention. They called for immediate government intervention to ensure the functionality of health centers, address workforce shortages, and guarantee a steady supply of IPTp-SP. According to the WHO [56], an effective healthcare system relies on skilled and motivated HCPs, well-maintained infrastructure, and a consistent supply of essential medications and technologies. Improving healthcare facilities is critical to ensuring the delivery of quality maternal healthcare services, particularly for pregnant women in rural and underserved areas [57-58]. This insight underscores a more extensive ongoing discussion on the pivotal role of both federal and state governments in strengthening public health systems and ensuring universal access to essential healthcare services and medications.

### Strengths, limitations, and future directions

This study's strengths include its socio-ecological approach, which examines IPTp-SP uptake across multiple levels, and its community-driven methodology, ensuring culturally relevant recommendations. The consistent and efficient data collection and transcription across multiple local languages and dialects (Ijaw and pidgin English) facilitated a smoother analysis. Utilising research assistants familiar with the local context and English ensured that participants' perspectives were captured in a culturally sensitive way and aligned with the study's objectives. The use of Appreciative Inquiry (AI) promotes solutions-focused insights, while conducting interviews in local dialects enhances inclusivity.

However, limitations exist. First, the study’s focus on two rural communities limits generalisability to the entire country. Nigeria is socio-culturally diverse, and significant health differences have been reported between northern and southern Nigeria [50,59]. Hence, an intervention programme that improves one part of Nigeria may lead to the reverse in another. Second, language barriers posed a challenge as the interview guide and informed consent were not formally translated into local dialects, relying instead on research assistants' real-time interpretation, which may have introduced slight variations in phrasing. Third, self-reported data may introduce recall or social desirability bias, and excluding HCPs leaves gaps in understanding service delivery challenges. Fourth, considering the sample, the recommendations suggested in this study may not be holistic. Data from nationally representative surveys, such as the Malaria Indicator Survey, may direct policymakers to robust recommendations that can improve IPTp uptake.

Future research should expand to diverse settings, incorporate healthcare providers’ perspectives, and assess intervention effectiveness in improving IPTp-SP uptake. Quantitative studies evaluating IPTp-SP adherence rates and health outcomes could complement this qualitative research, offering a more comprehensive understanding of the issues.

## Conclusions

This study underscores the pressing need for a multi-level approach to address the barriers hindering pregnant women’s uptake of IPTp-SP. By fostering community-wide education campaigns, enhancing the training and awareness of HCPs and community figures, integrating TBAs into formal healthcare systems, and ensuring government support for healthcare infrastructure and resource availability, significant strides can be made in improving maternal and child health outcomes. Such comprehensive interventions hold immense potential to reduce the prevalence of MiP, bridging gaps in healthcare access and delivery, and ultimately contributing to global efforts in maternal health and malaria prevention.
